# Practitioners’ experiences with 2021 amendments to Canada’s medical assistance in dying law: a qualitative analysis

**DOI:** 10.1177/26323524231218282

**Published:** 2023-12-25

**Authors:** Eliana Close, Jocelyn Downie, Ben P. White

**Affiliations:** Australian Centre for Health Law Research, Faculty of Business and Law, Queensland University of Technology, GPO Box 2434, Brisbane, QLD 4001, Australia; Health Law Institute, Faculty of Law and Medicine, Dalhousie University, Halifax, NS, Canada; Australian Centre for Health Law Research, Faculty of Business and Law, Queensland University of Technology, Brisbane, QLD, Australia

**Keywords:** euthanasia, medical assistance in dying, medicolegal issues, physician-assisted suicide, right to die

## Abstract

**Background:**

In 2016, Canada joined the growing number of jurisdictions to legalize medical assistance in dying (MAiD), when the Supreme Court of Canada’s decision in *Carter v Canada* took effect and the Canadian Parliament passed Bill C-14. Five years later, Bill C-7 introduced several significant amendments. These included removing the ‘reasonably foreseeable natural death’ requirement (an aspect that was widely debated) and introducing the final consent waiver. Since Bill C-7 is so new, very little research has investigated its operation in practice.

**Objectives:**

This study investigates the experiences of MAiD assessors and providers regarding the Bill C-7 amendments. It explores implications for understanding and improving regulatory reform and implementation.

**Design:**

Qualitative thematic analysis of semi-structured interviews.

**Methods:**

In all, 32 MAiD assessors and providers (25 physicians and 7 nurse practitioners) from British Columbia (*n* = 10), Ontario (*n* = 15) and Nova Scotia (*n* = 7) were interviewed.

**Results:**

The analysis resulted in five themes: (1) removing barriers to MAiD access; (2) navigating regulatory and systems recalibration; (3) recognizing workload burdens; (4) determining individual ethical boundaries of practice and (5) grappling with ethical tensions arising from broader health system challenges.

**Conclusion:**

This is one of the first studies to investigate physicians’ and nurse practitioners’ experiences of the impact of Bill C-7 after the legislation was passed. Bill C-7 addressed key problems under Bill C-14, including the two witnesses requirement and the 10-day waiting period. However, it also introduced new complexities as practitioners decided how to approach cases involving a non-reasonably foreseeable natural death (and contemplated the advent of MAiD for persons with a mental disorder as a sole underlying condition). This study highlights the importance of involving practitioners in advance of legislative changes. It also emphasizes how the regulation of MAiD involves a range of organizations, which requires strong leadership and coordination from the government.

## Background

Medical assistance in dying (MAiD) in Canada was legalized federally in 2016 when the Supreme Court of Canada’s decision in *Carter v Canada* (*Carter*) took effect and the Canadian Parliament passed Bill C-14 in response.^1,2^ Since then, law and practice have evolved significantly. From the outset, academics and others argued Bill C-14’s narrowness was unconstitutional and contrary to *Carter*.^3,4^ Two constitutional cases challenged eligibility requirements, including that a person’s ‘natural death has become reasonably foreseeable’.^5,6^

In September 2019, one of those constitutional challenges was successful (the other had been adjourned indefinitely^7^). In *Truchon and Gladu v Canada* (*Truchon*), Justice Baudouin ruled that the reasonably foreseeable natural death (RFND) requirement violated the *Charter* because, among other reasons, it discriminated against persons with disabilities whose natural death was not reasonably foreseeable.^5^ The federal government did not appeal, and after several delays introduced Bill C-7, which received royal assent in March 2021.^8^

Bill C-7 enacted several amendments to the *Criminal Code of Canada* (summarized in Tables 1 and 2). These included changes to directly respond to *Truchon*, namely, removing RFND as an eligibility criterion, and a corollary change establishing two sets of procedural safeguards (‘Track 1’ for persons whose death was reasonably foreseeable and ‘Track 2’ for persons whose death was not). Bill C-7 also introduced amendments prompted by advocacy efforts and evidence about barriers in practice under Bill C-14.^9^ These changes included removing the 10-day waiting period for Track 1 patients, streamlining witnessing requirements for the written request and permitting two types of advance requests for MAiD in limited circumstances (a ‘final consent waiver’ and ‘advance consent for failed self-administration’). Finally, Bill C-7 temporarily excluded access to MAiD for persons with mental disorder as their sole underlying medical condition (MAiD MD-SUMC). The Bill included a sunset clause that would automatically repeal the exclusion on 17 March 2023 (subsequently postponed to 17 March 2024).^10^

**Table 1.** Bill C-7 changes to eligibility criteria in section 241.2 of the *Criminal Code*.

Bill C-14	Bill C-7
• Eligible or would be eligible (but for any applicable minimum period of residence or waiting period) for publicly funded health services. • Is at least 18 years of age and capable of making decisions with respect to their health. • Has a grievous and irremediable medical condition: - has a serious and incurable illness, disease or disability; - is in an advanced state of irreversible decline in capability; - that illness, disease or disability or that state of decline causes them to endure physical or psychological suffering that is intolerable to them and that cannot be relieved under conditions that they consider acceptable; and - their natural death has become reasonably foreseeable. • Makes a voluntary request for medical assistance in dying. • Provides informed consent to receive medical assistance in dying after having been informed of the means that are available to relieve their suffering, including palliative care.	Changes to eligibility are: • Natural death has become reasonably foreseeable is no longer a component of a grievous and irremediable medical condition but does determine which track of safeguards applies (see Table 2): - Track 1 is for persons whose natural death is reasonably foreseeable - Track 2 is for persons whose natural death is not reasonably foreseeable • Mental illness is not considered a disease, illness or disability (until 17 March 2024, when this exclusion is automatically repealed)

**Table 2.** Bill C-7 changes to procedural safeguards in sections 241.2(3) and 241.2(3.1) of the *Criminal Code*.

Safeguards under Bill C-14	Track 1 safeguards under Bill C-7: Natural death is reasonably foreseeable [Criminal Code, section 241.2(3)]	Track 2 safeguards under Bill C-7: Natural death is not reasonably foreseeable [Criminal Code, section 241.2(3.1)]
*Before a physician or nurse practitioner provides medical assistance in dying they must. . .*		
Be of the opinion that the person meets the eligibility criteria.	Unchanged	Unchanged
Ensure the person’s request for MAiD is in writing and signed and dated by the person after being informed they have a grievous and irremediable medical condition.	Unchanged	Unchanged
Be satisfied the person signed and dated the request before two independent witnesses who also signed and dated the request. A witness is not independent if they: - are a beneficiary under the person’s will; - own or operate a health facility where the person is being treated or resides; or - are directly involved in providing health services or personal care to the person.	Signs and dates the request before one independent witness who also signs and dates the request. A person who provides healthcare services or personal care as their primary occupation and who is paid to provide that care to the person requesting medical assistance in dying is permitted to act as an independent witness unless they are the MAiD assessor or provider.	Same as Track 1
Ensure the person has been informed that they may, at any time and in any manner, withdraw their request.	Unchanged	Unchanged
Ensure another physician or nurse practitioner has provided a written opinion confirming that the person meets all of the eligibility criteria.	Unchanged	Another physician or nurse practitioner has provided a written opinion confirming that the person meets all of the eligibility criteria. If neither the assessor nor the provider has expertise in the condition that is causing the person’s suffering, the MAiD assessor or provider must consult with a physician or nurse practitioner who has that expertise and share the results of that consultation with the other practitioner.
Be satisfied they (i.e. the MAiD provider) are independent of the other physician or nurse practitioner (i.e. MAiD assessor).	Unchanged	Unchanged
Ensure there are at least 10 clear days between when the request was signed and MAiD is provided (unless the person is at imminent risk of dying or losing capacity).	Repealed	Ensure there are 90 clear days between when the first assessment begins and when MAiD is provided (this period can be shortened if both assessments have been completed and both the MAiD assessor and provider agree that the person’s loss of capacity is imminent).
If the person has difficulty communicating, take all necessary measures to provide a reliable means by which the person may understand the information that is provided to them and communicate their decision.	Unchanged	Unchanged
Immediately before providing MAiD, give the person an opportunity to withdraw their request and ensure that the person gives express consent to receive MAiD.	*Final consent waiver* This requirement for final consent can be waived before a person loses the capacity to consent to MAiD: • They met all eligibility criteria, and the Track 1 safeguards were met; • They entered into a written arrangement with the MAiD provider, indicating: - that the MAiD provider would provide MAiD on a specified day; - that they consented to the provider administering MAiD on or before the specified day, if they lost the capacity to consent to MAiD; and • The MAiD provider ensures they were informed of the risk of losing the capacity to consent to MAiD prior to the day specified. In these circumstances, the provider can administer MAiD under the terms of the arrangement provided the person does not refuse or resist administration (by words, sounds or gestures). *Advance consent – self-administration* If a person self-administers MAiD and loses capacity (i.e. failed self-administration), a MAiD provider can administer the substance, provided they entered into a written arrangement that the MAiD provider would: • Be present at the time of self-administration; and • Would administer a second substance if the person did not die within a specified period. In these circumstances, the provider can administer MAiD under the terms of the arrangement.	*Final consent waiver*Not available on Track 2*Advance consent – self-administration* Same as Track 1
N/A	N/A	Ensure that the person has been informed of the means available to relieve their suffering, including, where appropriate, counselling services, mental health and disability support services, community services and palliative care and has been offered consultations with relevant professionals who provide those services or that care.
N/A	N/A	Ensure that the MAiD assessor and provider have discussed with the person the reasonable and available means to relieve the person’s suffering and both assessor and provider agree with the person that the person has given serious consideration to those means.

MAiD, Medical assistance in dying.

Bill C-7 also mandated an independent expert review ‘respecting protocols, guidance, and safeguards to apply to requests made for medical assistance in dying by persons who have a mental illness’ and a comprehensive Parliamentary review of the MAiD law and its application, ‘including but not limited to issues relating to mature minors, advance requests, mental illness, the state of palliative care in Canada and the protection of Canadians with disabilities’.^8^ These review processes fuelled considerable public debate and significant media attention.^10,11,12^ Much of the public discourse centred on whether Track 2 MAiD was grounded in or exacerbated vulnerability for persons with disability and persons with mental disorders, particularly those with inadequate social support.^11–14^ Some argued that allowing persons to access MAiD solely based on mental illness or disability is problematic and that these persons should be excluded from eligibility for MAiD.^11–14^ Others rejected these conceptual/empirical claims and argued that excluding the choice to access MAiD is stigmatizing, paternalistic and discriminatory.^11–14^ Concerns were also raised about the vulnerability of Indigenous persons.^11,13,14^ However, submissions to the Special Joint Committee emphasized that empirical evidence from other jurisdictions fails to show that MAiD is problematic for vulnerable populations; more commonly, vulnerable individuals face barriers to access.^14^ Emerging data from Canada suggest a similar trend; most persons who receive MAiD are from relatively privileged social and economic demographic groups and have access to palliative care.^15–17^

In contrast to the vast debate about legal and policy aspects of Bill C-7, while there are some early studies,^18,19^ research on its actual operation in practice is still emerging (the Bill was only passed in 2021).^20^ Pesut *et al*.’s^18^ anticipatory study found nurses and nurse practitioners were concerned about disrupting the equilibrium that had been established post-Bill C-14, navigating complex Track 2 cases (‘a momentous change’) and responding to the lack of available resources for persons on Track 2.^18^ Wiebe *et al*.’s^20^ interviews with MAiD assessors and providers from January to May 2021 (conducted before and after Bill C-7 was passed) found that although unmet needs were perceived to rarely contribute to MAiD requests, complex chronic conditions led to ethical dilemmas about access to scarce resources. While Track 2 was a key focus of these anticipatory studies, federal data indicate that in 2021, just 2% of all MAiD deaths involved Track 2 (219 deaths).^16^ More data points will be available in 2024 when Health Canada provides data collected under new reporting regulations, which came into effect on 1 January 2023. Under the new regulations, clinicians are required to report on ‘preliminary assessments’ (which will expose reasons for finding people ineligible earlier in the process) as well as additional socio-demographic data including gender, race, Indigenous identity and disability (which will inform the understanding of who is requesting and who is getting MAiD).^16^

Another focus of the limited research on the implementation of Bill C-7 to date is the final consent waiver.^19^ The waiver allows persons whose natural death is reasonably foreseeable to waive the requirement that final consent is provided immediately before the administration of MAiD. This can occur only if prior to losing the capacity to consent to MAiD: the person meets all eligibility criteria; all other safeguards are met; and the person enters a written arrangement consenting to MAiD on or before a date specified in the arrangement. If the person loses capacity, the physician or nurse practitioner may administer MAiD according to the terms of the arrangement, provided the person does not resist or refuse the administration, by words, sounds or gestures. Research from Variath *et al*.^19^ found that although the waiver was perceived to support MAiD access, healthcare providers anticipated challenges including what date to set in the waiver, discomfort with invoking it when capacity is fluctuating or the family disagrees and concerns about a lack of resources to support implementation.

This paper seeks to add to the emerging literature on Bill C-7 by exploring the perspectives of MAiD assessors and providers regarding these changes to Canada’s *Criminal Code*. It aims to provide insights into the practical implications of the amendments on MAiD requesters, assessors and providers, and the provision of MAiD in Canada more broadly. This study sought to answer two key research questions: (1) What are the experiences and perceptions of MAiD assessors and providers regarding Bill C-7 (both before implementation of the changes with respect to MAiD MD-SUMC and post-implementation of the other C-7 changes)? (2) What are the implications of these experiences and perceptions for understanding and improving regulatory reform and implementation?

## Methods

This qualitative interview study is part of a broader international project that aims to develop an optimal holistic model of end-of-life regulation.^21^ This paper reports on data collected through semi-structured interviews with MAiD assessors and providers, namely physicians and nurse practitioners who assess patient eligibility for MAiD and/or administer the medication. The method is reported in accordance with the consolidated criteria for reporting qualitative studies (COREQ) (Supplemental Material 1).^22^

In Canada, while the *Criminal Code* is the responsibility of the federal Parliament, healthcare delivery is the responsibility of the provinces and territories and the mode of service delivery and regulatory context varies by jurisdiction.^23^ For feasibility, the study selected three target provinces: British Columbia, Ontario and Nova Scotia. These provinces vary in geography, population and frequency, and delivery of MAiD.^16^ Geographically, Ontario and British Columbia are large provinces, with the majority (over 80.0%) of the population living in urban areas. By contrast, Nova Scotia (like the other Atlantic provinces) is a small province with a relatively higher proportion of persons living in regional areas. By population, Ontario is Canada’s largest province (with 38.7% of the population in 2021), British Columbia is mid-sized (13.5%) and Nova Scotia is one of its smallest (2.6%). In Canada, in 2021 3.3% of all deaths were attributed to MAiD.^16^ Federal data indicate that British Columbia had the highest percentage of total deaths attributed to MAiD (4.8% in 2021), while Ontario and Nova Scotia had slightly less than the national average (2.7% and 2.5%, respectively).^16^ Another distinction is the MAiD delivery model varies between these provinces. In British Columbia and Nova Scotia, the majority of MAiD requests come through health authority care coordination services (68.3% and 57.5%, respectively, in 2021), and a minority come from the patient directly or other practitioners. By contrast, in Ontario, which has 14 different health networks with variable models of care, MAiD requests are split between three main sources: the patient directly (36.5%); care coordination services (41.6%) and other practitioners (18.5%).^16^

We sought participants who were involved in MAiD in these provinces as a MAiD assessor and/or provider. Under the *Criminal Code*, physicians and nurse practitioners are permitted to assess a person’s eligibility for MAiD and prescribe and/or administer the MAiD medication.^2^ The role of nurse practitioners in MAiD in Canada is unique; internationally, most jurisdictions with MAiD legislation rely solely on physicians to assess eligibility and administer the medication. As of October 2023, Canada is the only jurisdiction to allow nurse practitioners to do MAiD eligibility assessments [although some others, such as the Australian states of New South Wales, Queensland, Western Australia and Tasmania, permit nurse practitioners (and in some states, nurses) to administer the MAiD medication].

Calls for participants were distributed on social media (Twitter and the project website) and by the Canadian Association of MAiD Assessors and Providers (CAMAP) (the national professional organization for health professionals involved in MAiD). Participants were initially recruited using convenience sampling, based on who responded by email to these calls for recruitment, but purposive sampling and snowball sampling were used subsequently to enhance diversity based on sex, location and practice setting. Recruitment continued until there was adequate ‘information power’ to meet study aims.^24^

Data were collected using semi-structured interviews conducted by EC between 6 October 2021 and 18 October 2022 (with JD present for three initial interviews to refine the interview guide). The interviews were conducted using Zoom software (version 5.16.2, Zoom Video Communications, San Jose, California) (due to travel constraints imposed by the COVID-19 pandemic, and to facilitate the cross-Canadian sampling). EC used a semi-structured interview guide (Supplemental Material 2) which covered a broad range of topics related to MAiD decision-making for the broader study on regulation, mentioned above. EC raised the Bill C-7 changes using open-ended prompts if the participants did not bring them up themselves (e.g. ‘What are your views on or experiences with the Bill C-7 changes/Track 2/the final consent waiver?’). All participants were provided with a detailed participant information form and gave informed consent prior to the interview. Audio recordings of the interviews were professionally transcribed verbatim, and participants were allowed to amend their transcripts. EC maintained field notes and a reflexive journal throughout data collection and analysis and regularly debriefed after interviews with BW and JD.

Data were analysed using reflexive thematic analysis facilitated by NVivo software (release 1.6.1, QSR International, Burlington, Massachusetts).^25^ EC read all transcripts in full, identified all of the passages relating to Bill C-7 amendments and then inductively coded these extracts. EC developed initial themes from these codes, which were iteratively discussed and refined by all co-authors, with reference to the coding structure and dataset of passages relating to Bill C-7.

## Results

In all, 32 MAiD assessors/providers were interviewed (25 physicians and 7 nurse practitioners). Table 3 sets out participant demographics. Participants came from a range of specialties and practice settings in British Columbia (*n* = 10), Ontario (*n* = 15) and Nova Scotia (*n* = 7). Participants were a highly experienced group with a median of 20.5 years of experience in health care and 112.5 cases as MAiD assessor and/or provider. Interviews ranged between 50 and 203 min (median 98.5 min).

**Table 3.** Participant demographics.

Characteristic	Number of participants *n* = 32 (%)
Gender	
Female	21 (66%)
Male	11 (34%)
Age	
Median (interquartile range)	50.5 (42–61)
Province	
British Columbia	10 (31%)
Ontario	15 (47%)
Nova Scotia	7 (22%)
Population Centre and Rural Area Classification	
Large urban population centre (>100,000)	17 (53%)
Medium population centre (30,000–99,999)	5 (16%)
Small population centre (1000–29,999)	6 (19%)
Rural area	4 (13%)
Type of assessor/provider	
Physician	25 (78%)
Nurse practitioner	7 (22%)
Main clinical specialty	
Family medicine	14 (44%)
Primary care	6 (19%)
Palliative care	4 (13%)
Psychiatry	3 (9%)
Anaesthesia	1 (3%)
Geriatric medicine	1 (3%)
Internal medicine	1 (3%)
Neurology	1 (3%)
Oncology	1 (3%)
Practice setting	
Community only	16 (50%)
Hospital only	5 (16%)
Multiple settings (including community, hospital, hospice)	11 (34%)
Years of experience in healthcare	
Median (interquartile range)	20.5 (11–34)
Number of MAiD cases as assessor and/or provider	
Median (interquartile range)	112.5 (51–338)

MAiD, medical assistance in dying.

We identified five themes, summarized in Table 4.

**Table 4.** Description of themes.

Themes	Description
(1) Removing barriers to MAiD access	Participants discussed how some of the Bill C-7 changes (witnessing and the removal of the 10-day waiting period) were straightforward and removed problematic barriers to MAiD access in Bill C-14. Participants also thought other barriers were removed: ‘reasonably foreseeable natural death’ as an eligibility requirement, and the final consent waiver. They thought barriers created by Bill C-7 would be removed by the sunset clause for the exclusion of MAiD MD-SUMC. Participants noted aspects of the final consent waiver and Track 2 were challenging to implement and anticipated similar challenges when the exclusion of MAiD MD-SUMC was lifted.
(2) Navigating regulatory and systems recalibration	Participants discussed a process of recalibration responding to the Bill C-7 changes. Navigating regulatory and system changes brought similar challenges to when Bill C-14 took effect, with uncertainty about the scope of legal parameters, and some gaps in guidance. However, some participants emphasized that they were in a better position with Bill C-7 since they had an existing knowledge base about MAiD and established professional structures to draw on. Anticipating the end of the sunset clause, participants expressed concerns about the complexity of MAiD MD-SUMC. They sought more clarity about safeguards and best practices.
(3) Recognizing workload burdens	Participants highlighted additional workload burdens involved in implementing the waiver of final consent and Track 2 MAiD. The workload involved in supporting Track 2 patients and offering them appropriate referrals was a major factor that influenced practitioners’ willingness to be involved in these cases. The level of remuneration was insufficient for the work involved in Track 2. Participants flagged these burdens, particularly in relation to MAiD MD-SUMC cases.
(4) Determining individual ethical boundaries of practice	Participants described contemplating their willingness to be involved in cases involving patients on Track 2 in MAiD MD-SUMC. While some participants were willing to participate in these cases, others indicated they were not comfortable with them, for a variety of reasons including complexity, emotional load and ethical discomfort. Some participants described being willing to take on some types of Track 2 cases but not others.
(5) Grappling with tensions caused by broader systems challenges	Participants grappled with ethical tensions arising from limited health and social services resources and voiced concerns about the sustainability of and access to the MAiD system. There were also concerns about the impact of Track 2 and MAiD MD-SUMC on health system resources.

MAiD, Medical assistance in dying; MAiD MD-SUMC, MAiD where mental disorder is the sole underlying medical condition.

### Theme 1: Removing barriers to MAiD access

Participants perceived that Bill C-7 removed ‘unintentional’ (P24) barriers to MAiD access (see Table 5 for additional illustrative quotes). There was an overwhelming consensus that removing the 10-day mandatory waiting period for Track 1 patients and streamlining witnessing requirements were positive developments, addressing identified problems in practice. Participants also described the final consent waiver, Track 2 and the MAiD MD-SUMC sunset clause as removing barriers, though, as discussed in the other themes, these changes also introduced challenges.

**Table 5.** Theme 1: Removing barriers to MAiD access (additional example quotes).

Subtheme	Example quotes
Global impressions	‘I think the balance is good now, you know, with C-7. I think with C-14 there were definite barriers. C-7 I think got it right. I don’t think the safeguards are barriers anymore, but they don’t make it extra easy either, right. So, it hasn’t changed the eligibility criteria, but it took away what was actually unintentional barriers’. (P24) ‘I like the way Canada has done it incrementally. . . . the pipedream in the perfect world would be having iterative openings of the legislation, developing clinical practice guidelines, creating the infrastructure for healthcare providers like CAMAP, before the legislation comes out, and just everyone getting together and talking and listening and understanding and being tolerant’. (P10)
Streamlining witnessing requirements	‘It works way better now with C-7, in terms of only one witness and that witness can be a healthcare provider. It’s sort of a game-changer. It’s made it so much easier to get it done [the written request]. So before I would have said it’s a huge pain, it also penalizes people who have communication difficulties, who are isolated, who live alone. . . . people had trouble finding someone. They didn’t want to tell people, they wanted to maintain a sense of privacy . . . or they had shame around it. . .’ (P15)
Removing the 10-day waiting period	[There was] definitely a lot of anger [about the 10-day waiting period] from patients, like, ‘All this time no one told me how to do this process. Now I did the process finally and now I need to wait. Whereas if I had have just done this at the beginning or if someone listened to me a month ago, I would have never had to wait’. (P25) ‘. . .we were getting to the point where I think close to 50% of times we were waiving the 10-day reflection period because of. . . [fears about] . . . loss of capacity . . . it didn’t mean anything. And so it’s much, much better [now]. . .’ (P21) ‘. . .taking the 10 days away, [removed a barrier] which was just artificial and ridiculous . . .so many patients who presented with MAiD, it’s not that they thought about MAiD and called us right away. They’d been thinking about it for a bit. That was my beef about the 10 days’. (P31) ‘I liked the 10-day mandatory reflection period because I did think it was important for people to pause and make sure this was what they wanted to do. It also gave time for families to come together and kind of have some moments together. . . . [removing it] puts some extra demands on the providers. I mean those are totally my values . . . but I thought there was some utility there in that 10-day reflection period’. (P27)
Introducing the final consent waiver	‘I find that waiver fantastic. . . . I wish we had it three years ago. . . . I’ve had that happen so many times before the waiver, where they lost capacity and I couldn’t help them. Many, many, many times, many times. And their worst fear[s] . . . all these things they were terrified of happening happened. And I would just feel helpless and awful and the family’s distraught and, ugh, it’s awful. But now we have the waiver’. (P3) ‘. . .the waiver of final consent is mostly a good thing. It’s a good thing for patients and an irritating thing for clinicians [because of the challenges in interpreting and implementing it]. I think it gives patients a sense of greater autonomy, it calms their fears of losing capacity. Because there are so many cases where like people’s final days is racked with this fear of losing capacity instead of, you know, living their lives as they wish. So I think that in itself will always make it a worthwhile legislative change and one that I will always support’. (P15)
Removing RFND as an eligibility criterion and introducing Track 2	‘. . . just looking globally, I think we do have a nice balance. . . .from a patient level I think the focus has been less on that medical need to have a terminal illness, but it’s allowed again to focus on kind of suffering and the Track 2 overall. And getting rid of some of that really grey language [reasonably foreseeable natural death] that really mucked a lot of us up in the beginning, that’s been helpful as well. I’m much happier now with the balance than I think I was at the beginning. . .’ (P19) ‘I think it’s an improvement because it allows more people to have MAID that want it. But I think that it also allows some people that probably shouldn’t be getting it to, you know, come forward. Some of them get quite difficult when you tell them that you don’t think that they qualify at the present time’. (P4)
The MAiD MD-SUMC exclusion and sunset clause	‘In the previous language [under Bill C-14] mental health was not excluded. The criteria was such that you were pretty much never going to get anybody to pass on mental illness alone . . . [except for] anorexia which could fit. . . . You remove reasonably foreseeable, all of a sudden mental health is wide open. So they put in that piece in the bill ‘this is not an illness for the purposes of criteria A’, which is incredibly stigmatising and discriminatory . . . nowhere in Canadian legislation should there be the words ‘mental illness is not an illness’, no matter what the context is. . . . in the end we got the sunset clause in which is great’. (P10) ‘. . . I 100% believe it should be permissible for people with mental illness. But yeah, it’s just a question of how do we go about those assessments. And then if we decide that mental illness is different somehow from physical illness, we end up in this really thorny territory of the physical illnesses that are potentially actually mental illnesses’. (P7)

MAiD MD-SUMC, MAiD for persons with mental illness as their sole underlying medical condition; RFND, reasonably foreseeable natural death.

#### Streamlining witnessing requirements

Participants universally supported changes to witnessing requirements (described in Table 2). Participants thought requiring two independent witnesses was an ‘unreasonable barrier’ (P22) under Bill C-14. The ability to now use a single witness, who could be a health care provider, streamlined and improved the process for patients: ‘a game-changer’. (P15)

#### Removing the 10-day waiting period

Removing the mandatory 10-day waiting period for Track 1 patients also eliminated an ‘arbitrary [barrier]. . . and an artificial safeguard’. (P19) Participants often waived it because of imminent death or loss of capacity: ‘I waived it so many times. People who weren’t going to have 10 days’ (P11). Some thought that the waiting period was not needed for Track 1 patients, as their death was reasonably foreseeable, and patients had thought about MAiD extensively before completing a written request. Often, the waiting period just prolonged patients’ suffering.

This change was regarded as positive by nearly all participants; however, one participant preferred the 10-day waiting period because it gave families time to process the decision and reduced the urgency on practitioners.

#### Introducing the final consent waiver

The final consent waiver addressed another barrier to MAiD access for patients who met the eligibility criteria but lost capacity before MAiD could be provided. Some participants described difficult cases under Bill C-14, which were upsetting for families and stressful for providers:

I’ve had that happen so many times before the waiver, where they lost capacity and I couldn’t help them. (P3)

Many participants thought the waiver promoted patient autonomy and supported it as a narrow form of advance request. The waiver often provided comfort to patients who feared they may lose capacity and therefore access to MAiD.

Although participants viewed the waiver as a positive development (e.g. ‘fantastic’, P3), many voiced challenges in implementing it in practice (discussed in the other themes). Some participants, therefore, offered qualified support: ‘[it is] mostly a good thing’ (P15).

#### Removing RFND as an eligibility criterion and introducing Track 2

All but one participant thought removing RFND as an eligibility criterion was a positive step, in principle, which removed a barrier to MAiD access. The MAiD provisions in the *Criminal Code* were intended to provide choice to persons who were suffering intolerably, and these participants emphasized that the inclusion of Track 2 better achieves this:

. . .it was really unfair to . . . [exclude someone] if you are suffering as much as anybody else. (P24)

However, while most participants noted they supported the advent of Track 2 in principle, there was a range of perspectives regarding willingness to participate in Track 2 cases (discussed in Theme 4). This was driven primarily by participants’ ethical discomfort with providing MAiD to persons whose natural death is not yet reasonably foreseeable and/or challenges regarding implementation:

. . .do I believe that MAID should be available to these patients? Yes. But how do we sort out the pieces, how do we make that work? (P21)

Several participants acknowledged the tension between their in-principle support and refusal to participate (explored further in Theme 4):

. . .it’s a difficult tension for me, because . . . I don’t think I want to provide MAID beyond my current scope [Track 1]. I’m happy for someone to do it, but I don’t want it to be me. So I think many of us exist in this tension where we’re happy for it to be as liberal, you know, as it can be, but at the same time there are limits to our own personal comfort zone and it does require us to set certain boundaries. (P15)

The one participant who did not support the removal of RFND as an eligibility criterion stated:

. . .I would prefer it to be more confined to someone where there’s a foreseeable reason they’re going to die in the next little while. . . (P28)

#### The MAiD MD-SUMC exclusion and sunset clause

Several participants noted that although mental health was not excluded under Bill C-14, the RFND eligibility criterion had made it very hard to access MAiD with a mental disorder as the sole underlying condition. Bill C-7 removed RFND as an eligibility criterion but kept it as the determining factor for whether a person would be subject to Track 1 (RFND) or Track 2 (not RFND) procedural safeguards. With the removal of RFND as an eligibility criterion, more people with mental disorder as a sole underlying condition would have been potentially eligible for MAiD (albeit most often under Track 2 procedural safeguards). The exclusion of MAiD MD-SUMC under Bill C-7 therefore created a barrier:

. . . now I don’t think that we have unreasonable barriers, except for the mental illness one [until the sunset clause is lifted] which is wrong. (P22)

In contrast to the broad ethical support for non-MD-SUMC Track 2 cases, participants had varying levels of comfort with the concept of MAiD MD-SUMC. Several noted that excluding MAiD MD-SUMC was discriminatory and the distinction between physical and mental illness was not always clear. However, others expressed discomfort, and like current Track 2 access (i.e. with MAiD MD-SUMC prohibited), regardless of their degree of ethical support, participants had some concerns regarding implementation. One participant indicated they preferred the structure for MAiD MD-SUMC under Bill C-14, but understood why the law changed as it had:

I think the way that it was structured in C-14 where mental health patients could – that were not excluded, but they never really qualified except for very rare cases, was not a bad thing, to be honest. . . . I understand why it [the law] changed this way, I understand the mental health issue, and why it’s excluded, why it’s going to sunset. I think it all went through that process for the right reasons and ended up in the right place. (P8)

Another participant discussed the challenges of assessing the ‘grey’ areas:

. . . if you’ve ever seen a person who’s struggled with mental illness for decades and has tried everything, like how can you say that’s unreasonable? . . . the tricky part is those in-betweeners, right, and the grey areas. (P20)

These challenges impacted participants’ willingness to participate in MAiD MD-SUMC cases (discussed in the other themes).

### Theme 2: Navigating regulatory and systems recalibration

Participants described navigating regulatory and systems recalibration after Bill C-7 (see Table 6 for additional illustrative quotes). As noted in the Method section, Canada’s MAiD law is set at the highest level through the *Criminal Code*, which is under the jurisdiction of the federal Parliament.^9^ Implementation is then regulated through court cases and is regulated and systematized at the provincial/territorial, regional and local levels through provincial/territorial governments, regulatory bodies, MAiD programs, healthcare institutions and communities of practice. This theme reflects participants’ integration of Bill C-7 into practice, as well as consequential alterations to other regulations and system guidance.

**Table 6.** Theme 2: Navigating regulatory and systems recalibration (additional example quotes).

Subtheme	Example quotes
Uncertainty about the law	*Waiver* ‘But what if they’re fully eligible and the patient isn’t ready in their mind and they want a date a year from now? . . . you know, there’s nothing in the law that stops that, but then you have to say, “I’m not really comfortable with that. I’d like to just do this.” . . . then you’re trying to explain to the patient that “It’s actually not illegal. I just don’t want you to do it”’. (P19) ‘If I was revising that waiver, I would say this is for a time-limited sense for something that’s going to occur in the next 10 days, you know, and have it more time limited. Then who are the persons delegated to inform me of the person’s loss of capacity. . . .I would specify people’. (P12) *Track 2* ‘Have they seen the relevant specialists? Have they been offered the relevant types of care? Is it accessible? You know, it’s all very well saying this patient should have CBT [cognitive behavioural therapy]. If CBT is not available, that’s an issue. Is it okay for somebody to say, ‘Give me CBT and I’ll stay alive, but don’t and I’ll die,’? Those are the sorts of things that Track 2 assessors and providers are tackling now. . . . This stuff doesn’t exist in Track 1. These problems don’t exist, or at least they hardly ever did’. (P6) *MAiD MD-SUMC* ‘Someone made the argument that, ‘Oh, mental illness can always get better. You can never call it irremediable. There’s all these new treatments coming out’. . . .[but] you can’t keep asking people to wait for the next new thing that’s barely been researched and shows promise. You wouldn’t say that for heart failure’. (P20) I think one of the biggest challenges is around the irremediability . . . as a . . . clinician with that patient sitting in front of me, I find myself thinking I absolutely don’t know if, for example, this person’s depression is remediable if they’ve only tried two or three treatments or if they’re never tried ECT or if they’re never tried therapy. . . . As an assessor I probably do need some guidance around how to define irremediability . . . the question is what’s the level of uncertainty that we’re willing to tolerate. . . (P7)
Inadequate guidance	‘I didn’t even hear about the waiver until July. I was like, “Oh, that Audrey’s amendment thing? Like, oh, that happened. Oh, did it? Oh, last March? Oh, my goodness.” Yeah, like terrible. . .’ (P26) ‘Oh, no, much more [support] with C-7, much more. Because, you know, CAMAP was in place. It was a much, much better process. We already – we had talked about C-7 coming into effect for years, so we were all prepared for C-7, you know, conceptually’. (P21) ‘CAMAP has provided some guidance articles or guidelines. I haven’t really needed them because I’ve been doing this for long enough. Like the change isn’t huge in terms of the eligibility criteria or anything. You know, the change more creates a few logistical challenges, but it doesn’t change much beyond that’. (P20) ‘. . .initially it was quite troublesome because there was no guide for this and there were no forms available. So basically I just wrote a handwritten note saying, “I, so-and-so, understand I’ve been qualified to have MAID and I would like to have it on such-and-such a date. However, in the event that I lose capacity before that time, I would still like it to proceed.” I get them to sign it and date it and I get a witness. There’s now a form in some of the provinces which has made it much easier’. (P4)
Sources of guidance	‘. . . our MAID team did a very good job of updating all of us that were on the current provider and assessor list. You know, they provided us with resources, they provided us with education sessions around what the changes were. For someone who doesn’t have a great coordination and resource centre like we do, I’m sure that it was all self-taught and hope you got it right’. (P16) ‘. . .only from CAMAP. CAMAP has done a number of symposia and webinars, and we have our case sharing webinars monthly so, again, that’s the forum in which we explore these, and CAMAP is doing a fall symposium right now where they are specifically looking at some of the changes in the legislation to see how they are working, but that’s an initiative that comes from the clinicians themselves, not from any medical body or supervisory body’. (P5) ‘I think the only other things that really played a role was the subsequent court cases. So we had . . . *AB v Canada* to talk about what is reasonably foreseeable death, a case in Nova Scotia where a wife tried to prevent the husband from having MAID and . . . they said, “You can’t interfere with a spouse’s choice,” and eventually the *Truchon, Gladu* and [the] Julia Lamb cases. So all those cases and their outcomes determined how we changed practice, and not expanded scope but, actually, were more safe to actually follow the liberal interpretation of the law. Because before . . . We weren’t quite sure what’s allowed, what’s not allowed’. (P24) ‘It is something that by its nature is so divisive and so uncertain [MAiD for Track 2] and so unknown that it takes a year or two before I think there is that, okay, now we get it. Now we know what expertise is. Now we know that, yeah they actually don’t have to have been to six chronic pain clinics, it’s okay, but it takes people willing to put themselves out’. (P9) ‘. . .every clinician, as they gain experience, changes. And so that’s what I remind people of. And when they say, “Well, what’s going to happen with this new law?,” right, then I say, “Well, think back to the first day you graduated. The rule for whatever might be exactly the same as it is today, but your interpretation of how to manage even somebody’s blood pressure might be different because you’ve gained experience to go with that.” So that’s where I think we’ll learn over time, and how are practice will expand with our experience’. (P19) ‘So even though the date is coming and looming [for MAiD MD-SUMC to take effect], I think it will have to be a soft start out of necessity. I think it will be a smattering of psychiatrists doing that work and kind of – just like MAiD started, I think it’ll be similar. Very few people doing it until we start to get a better understanding of all those things we never thought we needed to know. Once we realise that, I think we’ll start having a better idea on how to roll that out’. (P25) ‘You invest on the development of that practice guideline. It drops two months beforehand. Everyone needs to know it. The Colleges put in regulations that you have to follow this clinical practice guideline when doing MAID. Then everything comes up and the legislation comes up and it’s kind of barebones, that framework, but you’ve got the regulatory body already there with the teeth in place to back up the clinical practice guideline that’s already been created by all the key stakeholders. That’s how I would like to see it happen’. (P10)

#### Uncertainty about the law

The lack of clinical specificity in some *Criminal Code* provisions in Bill C-7 led to uncertainty about the law. Many participants found this uncertainty and resulting variation in practice challenging. Regarding the final consent waiver, one participant noted it was:

. . . the most important change in C-7. . . . the most straightforward, the most obvious thing we needed to do, everybody agreed . . . It’s turned out to be oddly more confusing and more difficult to use than people ever expected in terms of both clinicians and patients. (P8)

Some participants were uncertain about aspects of the waiver including whether it required a witness, what needed to be documented and the date for provision. Some participants thought discretion regarding the date was appropriate, whereas others sought more clarity and certainty about appropriate boundaries. Several participants mentioned that setting a date too far in the future (e.g. 6 months or more) effectively turned the waiver into ‘a form of advance directive’ (P15), which was not in ‘the spirit of the legislation’ (P15).

Regarding Track 2, several participants discussed the challenges of interpreting RFND. After Bill C-14, there was significant debate about the meaning of RFND (which is not defined in the *Criminal Code*) and how close to death a person needed to be to meet this requirement.^3^ On a narrow interpretation, a person has an RFND if their death is expected to occur within a time period that is not too remote (case law confirmed that RFND did not require practitioners to estimate a specific timeframe). Interpreted more broadly, an RFND can also apply to a person who has a predictable cause of death, such as a diagnosis with a lethal condition (i.e. without the temporal link). Although Bill C-7 removed RFND as an eligibility criterion, participants emphasized they still had to interpret RFND under Bill C-7 since it determined the track of safeguards that applied (Track 1 for persons with an RFND and Track 2 for persons without an RFND). They again reported variation:

. . .those of us that . . . want to continue to work with the same definition post-legislation change are being challenged by others who think it should now be interpreted much more narrowly, but that creates a problem because if you interpret it narrowly now then what were we doing before? (P5)

Participants were also uncertain about some of the Track 2 safeguards (see Table 2 for an overview), including the requirement that if neither assessor or provider has expertise in the condition causing the person’s suffering, the MAiD assessor/provider must consult a physician or nurse practitioner who has that expertise. Participants queried who has the requisite expertise for complex chronic conditions:

. . .[if] it’s a disorder that can affect . . . a whole bunch of systems . . . who do I consult? (P27)

There was variability regarding the expertise requirement, with some participants always looking to refer and others indicating they would often gain the expertise if they did not have it:

. . .the way I have dealt with it, which is not the way other people have dealt with it at all, is to get the expertise myself. (P22)

Another challenge was the requirement to inform Track 2 patients about the means available to relieve their suffering and offer them consultations with the relevant professionals, including, where appropriate, counselling services, mental health and disability support services, community services and palliative care. Participants needed to navigate determining: (1) the range and volume of consultations for complex chronic conditions; and (2) what constituted adequate referrals in an environment of scarce resources (discussed further in Theme 5). These uncertainties were novel:

This stuff doesn’t exist in Track 1. These problems don’t exist, or at least they hardly ever did. (P6)

Regarding MAiD MD-SUMC, participants were concerned about how to determine the capacity and irremediability of a mental disorder. Participants thought mental health conditions could be irremediable but wanted more guidance on how to establish this and described burdens as a clinician to make this call. Participants also discussed concerns about how to judge whether a person’s mental illness compromises capacity for MAiD.

#### Inadequate guidance

Several participants likened the implementation of Bill C-7 to the period after Bill C-14 came into effect, with little time for preparation and a lack of immediately available guidance. One participant commented:

We thought between the passing with the vote and royal assent we might get a day or two to get organized, and it was 10 minutes. (P19)

Navigating new laws and their implementation requires guidance from provincial/territorial governments or other regulatory bodies but several participants emphasized this did not occur: ‘no formal release of much of anything’ (P18); ‘It’s zero’ (P8). Others noted there was ‘much more with C-7’ (P21), including information from provincial Colleges, local health authorities and MAiD teams in healthcare institutions. Some noted that the implementation of Bill C-7 was smoother than for Bill C-14 because of existing knowledge and experience with MAiD and established professional supports, including CAMAP. Some participants found the lack of guidance about Bill C-7 stressful, while others noted that they did not need much guidance as they had considerable MAiD experience:

. . .the change isn’t huge in terms of the eligibility criteria or anything . . . [it] creates a few logistical challenges, but it doesn’t change much beyond that. (P20)

Participants mentioned that final consent waiver resources varied by location and practice setting (e.g. community *versus* hospital). British Columbia and Nova Scotia had developed a waiver form, while Ontario had not. Several participants in Ontario would have valued proactive guidance and waiver forms from the provincial government:

. . . if I’m using the MOH [Ministry of Health] form, they sure can’t argue that I left information out. (P9)

Again, some participants found this lack of guidance about the waiver was stressful. One participant worried about the effect on less experienced practitioners:

They shouldn’t be leaving this up to us as individual clinicians. I think it’s really messed up that we have different interpretations of this [the date in the waiver]. . . . as a more experienced provider I don’t feel that anxiety around the uncertainty or the different ways people are doing things, but I think it’s really like unfair to new providers and really hinders our ability to recruit new providers when these rules aren’t clear. (P20)

Several participants created their own form (some relying on precedents from other provinces and/or from Dying with Dignity); one said this was ‘troublesome’ (P4) because of inadequate guidance from the Ontario government. A few participants sought consistent guidance and practice standards regarding the waiver across the country.

Participants also noted guidance will be needed for MAiD MD-SUMC when the sunset clause takes effect, including guidance for assessing irremediability and capacity. Participants also wanted to know the parameters of clinical practice standards for MAiD MD-SUMC well in advance of this aspect of the legislation taking effect (a concern that may now be addressed, at least in part, by the subsequent release of a Model Practice Standard developed by an independent expert group and published by Health Canada^26^). One participant discussed the importance of timely standards enforced by College regulations.

#### Sources of guidance

Participants described various sources they relied on to navigate Bill C-7, including the legislation; CAMAP webinars and information sessions; advice from the Canadian Medical Protective Association, the Canadian Nurses Protective Society (professional liability risk management providers and insurers) and key legal academics; information from healthcare institutions, local health authorities, MAiD coordinators and their College (provincial regulatory body); provincial forms and guidance (where available); and colleagues (both informally and through communities of practice, such as the CAMAP listserv).

Many participants indicated that CAMAP provided the most comprehensive information, and thought that guidance developed by clinicians, rather than material from federal or provincial/territorial government or other regulatory bodies, was most relevant. CAMAP case sharing, symposia and webinars were described as:

. . .an initiative that comes from the clinicians themselves, not from any medical body or supervisory body. (P5)

As a professional organization, CAMAP was perceived as driving high-quality MAiD practice because it is:

. . .into excellence and always providing the best care and helping each other and supporting each other. . . . Governments don’t do that, and we don’t expect or want them to do it. (P22)

Participants also emphasized that exercising clinical judgement was essential. Their individual and collective understanding of the legislative terms developed through experience over time (as with all medical practice):

. . .now that I’ve done dozens of them, I now have a way of approaching it that kind of makes sense. (P29)

A key part of this developing understanding was individual clinicians who took on complex cases and thereby figured out how to interpret and apply the legislation. Other key sources of guidance were case discussions with colleagues both informally and within communities of practice (both CAMAP’s and within institutions and local authorities) and court cases, which refined clinicians’ understanding of the law.

### Theme 3: Recognizing workload burdens

Participants discussed workload burdens arising from the waiver and Track 2 (see Table 7 for additional illustrative quotes).

**Table 7.** Theme 3: Recognizing workload burdens (additional example quotes).

Subtheme	Example quotes
Logistics of the waiver	‘I generally don’t bring it up [the waiver] as something that I say to all of my patients [unless they are concerned about losing capacity]. Whereas I know there are clinicians who tell all of their patients . . . but . . . that can become very onerous’. (P1) ‘I think there are challenges with it [the waiver]. . . . it adds paperwork, for sure, and . . . [is challenging when] someone wants the waiver signed but they are delaying MAID for a certain amount of time. So the question is how often should you have that waiver re-signed, how frequently should that be confirmed? So there are a lot of questions about that among providers. . . . some guidance would be helpful for providers about how long – when does that expire’. (P7) ‘. . . not everybody has the capacity to sign a waiver. It’s a document with six or seven terms and conditions. And I think there’s a difference between having capacity for MAID and a capacity to sign a waiver. I think they’re two different things. . . . I will not sign a waiver with somebody who I do not believe has the capacity to understand all those terms and conditions’. (P21)
Track 2 cases are onerous	‘So those cases are definitely more onerous. They are extremely time consuming. A lot of our providers who have I’ll call it MAID as a “secondary duty” do not take these patients on. Oftentimes these patients don’t have a care provider, most oftentimes because they’ve alienated their care provider because of – it’s just the nature of the disease process, right, that a lot of people under not reasonably foreseeable are seeking death’. (P16) ‘I’m just not comfortable and I don’t have the capacity or the time to manage all of the work that’s going to have to happen for this assessment. It’s going to take days, you know, to get all these things and all the phone calls. And often, you know, for NPs [nurse practitioners], we don’t necessarily have the support of administration or anything in the background. So as an NP in the community you do everything yourself. You make your own phone calls yourself, you do everything, and that will eat up all of your time’. (P19)
Insufficient remuneration	‘. . .just practically speaking, from an assessor perspective, we don’t have a fee code or a billing code that allows us to have those detailed interactions with people [Track 2]. So if I’ve had five or six interactions with a person going through this period of time, I billed for the assessment once’. (P32) ‘I agreed to be a MAID physician, I didn’t want to take on this patient as my primary responsibility for three months. So that is (a) not compensated, and (b) it may not be what you’ve signed up for’. (P12)

#### Logistics of the waiver

The waiver added paperwork, but more significantly it increased the complexity of discussions and required practitioners to choose who to offer it to. Some participants completed a waiver with every Track 1 patient, reasoning:

. . .anyone who has a reasonably foreseeable death is at risk of losing capacity for one reason or another, whether it’s escalating narcotic dosing, whether it’s possibility of stroke from a metastatic process. (P16)

Others noted completing a waiver with every Track 1 patient could ‘become very onerous’ (P1), and only completed it for a subset of patients, for example, those with fluctuating delirium or brain cancer. Another unexpected area of complexity mentioned by a few participants was some Track 1 patients can consent to MAiD but not understand the more complex concept of picking a specified date and consenting to MAiD on or before that date. For example:

The trouble is implementing it. So I don’t offer it to them – because sometimes I feel like patients can have capacity to consent to MAiD and not actually have capacity to consent to the waiver. (P19)

#### Track 2 cases are onerous

Track 2 cases were rare and arose less frequently than was anticipated:

. . .people were prepared for an onslaught and there just wasn’t any. . . But the single cases are extremely hard and extremely burdensome in terms of volume of work. (P24)

Track 2 cases were ‘time-consuming’, ‘onerous’, ‘difficult’, ‘challenging’ and ‘very rarely straightforward’ (e.g. see Table 7) and added significantly to practitioners’ workload. These cases were more complex than Track 1, which led MAiD assessors and providers to make trade-offs:

I can see 20 patients that need my help before I can see one of these [Track 2] patients. (P19)

Some participants had no experience with Track 2, while other participants had seen multiple Track 2 patients. Some Track 2 cases were more straightforward (e.g. spinal cord injury), while more difficult cases involved complex chronic conditions (often with comorbid mental health conditions), such as chronic pain. Additionally, the required offering of consultations for Track 2 patients was time-consuming for patients without a primary care provider (e.g. general practitioner) to facilitate this (often due to a shortage of primary care providers). One participant felt as if:

I’d become the person’s primary care provider. I’m now responsible for making sure that they’ve had access to all these services. . . . I’ve already got a full-time job. . . . This is something I do as an extra. (P9)

#### Insufficient remuneration

Related to workload was remuneration. Participants indicated they were not adequately compensated for the extra time involved in Track 2 cases and this was a central limiting factor for how many they could take on. For example:

. . .the question is always – if you got paid properly to do it, and for the time it really takes, you probably wouldn’t have an issue, but it’s because it’s also not paid properly and you don’t have the time to do it. (P24)

### Theme 4: Determining individual ethical boundaries of practice

Participants described determining their boundaries of practice after Bill C-7 and how to exercise their discretion within the *Criminal Code* (see Table 8 for additional illustrative quotes). This involved new ethical tensions in their MAiD practice.

**Table 8.** Theme 4: Determining individual ethical boundaries of practice (additional example quotes).

Subtheme	Example quotes
Ethical tensions regarding the waiver	‘. . . the other thing, too . . . is it’s really nice if all the family are on board with it. If there is someone who may even a little bit be opposed to MAID or the decision that the person is made, enacting a final waiver of consent can be a little bit daunting for a provider’. (P15) ‘. . .when my dementia patient [completes the waiver] and then they . . . lose capacity, and the question is when do I invoke that? How do I decide it’s time? I don’t need the family’s consent, but I’m not going to go ahead without the family’s consent. That’s a lot of responsibility. That feels much more cumbersome than MAiD in general because I’ve got a patient who’s sitting there and walking and talking, who’s no idea what’s going on and I’m supposed to put a needle in their arm and end their life. That’s much more tense. That’s much more difficult morally for me and I’m not quite sure if I’m going to feel I can actually do that’. (P8)
Determining individual scope of practice regarding Track 2	‘I think they’re rare [Track 2 cases]. . . . I’ve never indicated to anybody that I wouldn’t do them. So I think they are still quite rare. They’re not that common. . . . so far the one case I had was a chronic pain, but I had absolutely no doubts about that patient’. (P17) ‘There’s a comfort level [with Track 1] knowing that we’re just changing how they die, not whether they’ll die. So the further we get from that proximity, we’re really determining whether they’ll die or whether they’ll not die, and not how they’ll die. So that I think is a significant paradigm shift’. (P15) ‘I will see Track 2 patients, but I am really selective. . . .. So I am fine to see a paraplegic patient. . . . They’re not dying, they’re suffering, but it’s a definitive diagnosis. I really don’t want to see multiple chemical sensitivities or things that you can’t at all measure. I don’t want to see a syndrome. And I don’t feel clinically like I have enough experience to even know whether they’re real’. (P19) ‘I don’t think it’s unethical to say, “I’ll do Track 1s and not Track 2s”. They are so difficult, they are so emotionally challenging. It’s just, you know, we need to support those people that are doing it, that’s what we need to do’. (P21) ‘My ethics I guess are that if the public wants it [Track 2], who am I to say no? We are in a position as physicians – and nurse practitioners would be included in this – that we have a monopoly on care. So I think it’s inappropriate for us to decline patients things based on our own ethics’. (P20)
Determining individual scope of practice regarding MAiD MD-SUMC	‘I’d say ideologically I think everybody should have access. I’d struggle more with how I’m willing to participate in that. I very much support the idea that somebody who has mental illness . . . to have access. I think it’s so much harder to implement and police, so I struggle with that’. (P11) ‘The mental health aspect of things too, like that one makes me also a bit nervous. . . . I do find a lot of practitioners are starting to be like, “No, I’m kind of only comfortable in these two tracks. I’m not really comfortable in this”’. (P18) ‘I already don’t feel comfortable with the idea of mental health as the sole reason because I see mental health as being variable and frequently curable, or at the very least managed well. I have patients with severe schizophrenia that are happily married with kids on very high doses of medication, and have had episodes where they were suicidal, they were homicidal, where they failed to received adequate treatment. So those people, I don’t know if I would want to be involved in those cases’. (P26)

MAiD MD-SUMC, MAiD for persons with mental illness as their sole underlying medical condition.

#### Final consent waiver

Participants varied in approach and ethical comfort with their discretion regarding waiver date (perhaps also reflecting uncertainty in the law: Theme 2). Some only felt comfortable with a date 3 months in advance; any longer meant the patient saw MAiD as a ‘back pocket’ option rather than actively seeking it. By contrast, some participants were comfortable with up to a year in the future:

Well, I’m personally comfortable with up to a year so I allow my patients to do that. I’ve many colleagues that are only comfortable with up to three months. (P8)

Other participants fell in the middle. Given the law provided flexibility, when patients asked for a longer period than participants were comfortable with, some found this challenging to navigate.

Participants were also concerned about invoking the waiver, particularly with patients who had lost capacity but did not appear to be suffering. For example, one participant indicated she would not invoke the waiver for a patient with dementia whose suffering eased the more she lost capacity: ‘The law will permit me, but my inner moral compass won’t’ (P6). A related concern was the emotional toll:

I still don’t know how that will feel. . . . it is different having someone say ‘Okay, I’m ready’,. . . there is this sort of emotional difference. (P15)

A further concern was whether to invoke the waiver when the patient’s family disagreed or expressed discomfort. Although the law does not require family or substitute decision-maker involvement or consent to complete or invoke the waiver, participants indicated they found the prospect of family dissent emotionally and ethically challenging. For example:

So I have a contract with my patient who’s lost consciousness and the family says, ‘Please don’t do anything’. . . . I don’t feel like I can march in with drugs to an unwelcome household . . . So that’s another conundrum, right? (P12)

A few participants usually asked a family member or other close contact to sign the waiver form (even though this is not required by the legislation). This was perceived to help ensure the person signing was aware of the patient’s intentions, to reduce the likelihood of complaints or dissent and to create a mechanism to notify the practitioner if the person lost capacity.

#### Track 2

Participants described having to decide their level of participation in Track 2 cases given they were more complex, more time-consuming (often unremunerated), and emotionally more difficult. For some, there was tension between the more onerous nature of these assessments and the desire to help Track 2 patients. These participants did not want to abandon patients who were suffering yet had to draw boundaries based on their availability and emotional load. For example:

. . . [Track 2] patients are difficult . . . in that as a provider I have to be certain that I’ve gone every avenue and tried to do what I can for these folks so they don’t end their lives. And when there’s only so much that I can rationally and reasonably do, sometimes that’s a little bit hard on the psyche. (P16)

Another participant emphasized the challenging and unexpected nature of the Track 2 population:

. . .the majority of the requests are for chronic pain conditions and that’s not really what we were expecting. . . that has led to a lot of burnout and refusals among providers to accept Track 2 patients. (P7)

Others emphasized their ethical discomfort with providing MAiD for persons who are not dying, with one participant describing that it ‘feels to me like assisting suicide more than medicating a death’ (P26). Another participant commented:

I never had that feeling before Bill C-7. But I’ve had it several times since Bill C-7 thinking, you know, God, I’m not really sure that this is the right thing to be doing. (P4)

These challenges led some clinicians to refuse to take on any Track 2 cases, while others drew lines regarding the types of Track 2 patients they would see. For example, one participant was willing to see Track 2 cases with definitive diagnoses (such as paraplegia) but felt reluctant to assess complex and/or controversial syndromes (such as multiple chemical sensitivities) (see Table 8, P19).

Participants had varied views about their Track 2 scope of practice. Some participants felt confident not participating: ‘I know I don’t have the bandwidth to and I’m giving myself a break on that’ (P9). Others experienced discomfort with the clash between philosophical support for Track 2 MAiD and reservations about engaging in it. They questioned the ethics of MAiD assessors and providers picking and choosing their cases:

. . . if the public wants it, who am I to say no? I think it’s our obligation in a way to do these [Track 2] assessments. (P20)

#### MAiD MD-SUMC

Participants anticipated similar issues regarding participation for MAiD MD-SUMC once it became legal (with the automatic repeal of the exclusion clause). Analogous to their views on existing Track 2 cases, several participants indicated they did not feel comfortable with providing MAiD MD-SUMC, even though they supported it. Participants anticipated a lack of willing providers for MAiD MD-SUMC which could create problems for patient access:

. . .most of the providers I know have said, ‘Yeah, I think this is a good idea [MAiD MD-SUMC] but I’m not doing it’. (P19)

### Theme 5: Grappling with ethical tensions arising from broader health systems challenges

Participants discussed ethical tensions related to scarce healthcare resources and their allocation. They discussed: contending with access to MAiD in the context of limited health and social services resources; sustainability of and access to the MAiD system and the impact of Track 2 on the broader health system.

#### Contending with access to MAiD in a context of limited health and social services resources

Participants described contemplating whether a lack of access to supports and services factored into a patient’s MAiD request (see Table 9 for additional illustrative quotes). In some cases, participants perceived unmet needs contributed to suffering. For example, one participant described finding a prospective Track 2 patient ineligible because, in part, they felt that a lack of finances contributed to the patient’s suffering: ‘I hated that the finances were a part of it’ (P1). Other participants noted that unmet needs did not appear to be driving MAiD requests but did add complexity to the eligibility assessments and, for Track 2 patients, added responsibilities with respect to informing and offering consultations with additional players in the system. Some participants also emphasized the ‘hard reality’ (e.g. see Table 9, P16) that the health system has limited resources and these broader systemic issues arise with other healthcare decisions. These tensions with scarce resources led some practitioners to decline to participate in Track 2 MAiD.

**Table 9.** Theme 5: Grappling with ethical tensions arising from broader systems challenges (additional example quotes).

Subtheme	Example quotes
Contending with access to MAiD in a context of limited health and social services resources	‘Our resources are lacking, especially in mental health. So it becomes very difficult to connect people with mental health services in a timely fashion. So I do not believe that our healthcare system is meeting the needs of a lot of our C-7 patients. We do our best, but there are also the hard realities of where we are’. (P16) ‘. . . it becomes my responsibility to get them access to all these services. These are services that aren’t paid for by [provincial health insurance], social work, physio, all of those kinds of things, or there are two-year wait lists and I have no way to make that wait list go faster’. (P9) ‘. . .people shouldn’t have to have MAiD for anything because they couldn’t get care otherwise . . . I think that that may be one of the issues in our community. And so I’m not getting them a psychiatric consult for MAiD when I couldn’t get them a psychiatric consult for healing, for wellness, right’. (P23) ‘. . .how do you balance the fact that we’ll never fund a social structure that meets everybody’s needs? So then are they ineligible for MAiD because they don’t have the social structure? You know, it’s hard. . . . on an individual level . . . when you put a face to it you go, “They should have that.” But when you consider how many people [have unmet needs] and what would that do to the system as a whole, it’s not an easy answer’. (P19)
Sustainability of and access to the MAiD system	‘We just need a lot more MAiD providers and they need to be paid properly. But neither’s going to happen, so the system is going to get derailed. . . . there are going to be MAiD providers who recognise the ethical quandary of choosing to be a MAiD provider for some types of people and not others and will solve the problem by retiring’. (P6) ‘So for every ten Track 1s we do, we are encouraged to do one Track 2. But, really, it’s tough. And when there’s not enough access for the Track 1s, I think we need to prioritise that too. However, that’s unfair to the Track 2s, but . . . as an individual clinician I can’t solve that’. (P25) ‘. . .there’s whole chunks of this province, big chunks of this province where nobody will see Track 2 patients’. (P22) ‘I can understand how it’s going to be for mental health, because there are all sorts of psychiatrists who are not going to want to be involved in this process, the vast majority’. (P32) ‘There’s people with non-reasonably foreseeable deaths [Track 2] who have been waiting since March [8 months] for a first assessment, so like how, I don’t understand how we don’t have a legal obligation to find them somewhere else where they can be assessed, so those complex issues about ownership of the program really, I struggle with a lot’. (P19)
Impact of Track 2 MAiD on the broader health system	‘There is not a psychiatrist in [regional area] who will accept a referral [for any reason]. And out of area, if people are willing to travel, they won’t accept referrals out of area. And it’s true, people shouldn’t have to have MAiD for anything because they couldn’t get care otherwise, but I really – yeah, I think that that may be one of the issues in our community. And so I’m not getting them a psychiatric consult for MAiD when I couldn’t get them a psychiatric consult for healing, for wellness, right’. (P23)

MAiD, Medical assistance in dying.

#### Sustainability of and access to the MAiD system

Participants raised two major concerns about the sustainability of the MAiD system post-Bill C-7. First, many participants commented that Bill C-7 changes were not adequately resourced, particularly in some locations (e.g. rural and regional areas, community settings). Some worried that without proper resources Track 2 could threaten the sustainability of the MAiD system as a whole: ‘We don’t have enough [providers] for Track 1 let alone Track 2’ (P32). Some participants described individual providers shouldering most of the Track 2 burden, feeling obligated to take on more cases as the only ones doing them. There were concerns that Track 2 would lead to provider burn-out, cause some providers to retire and would deter new practitioners from becoming MAiD providers. Participants thought the integrity of the MAiD system needed to be protected through appropriate support and remuneration for practitioners willing to see Track 2 and MAiD MD-SUMC patients, but some were sceptical this would happen (e.g. see Table 9, P6). Education and tools for practice were perceived as being critical components to attracting, supporting and retaining MAiD assessors and providers.

Second, participants were concerned that the lack of willing providers would compromise timely access for Track 2 MAiD patients. Several participants noted that there were whole regions, and institutions, who would not see Track 2 patients (increasing burdens on willing providers to travel), and that wait lists were lengthy. Similarly, some participants thought a lack of psychiatrists and other resources (beyond assessors and providers) meant access to MAiD MD-SUMC would be very limited in practice.

#### Impact of Track 2 MAiD on the broader health system

A few participants also voiced concerns that Track 2 MAiD patients could shift ancillary healthcare and social services resources away from others in the broader healthcare system. Participants felt conflicted that people might use a MAiD request to gain access to scarce resources, such as psychiatry and housing, before others. One participant noted:

. . .in this region at least, they said we’re not letting them queue jump. We have a two-year wait for chronic pain and they [the chronic pain clinic] said, ‘. . .MAID can’t be a way to queue jump’. (P31)

Similarly, some participants noted that they were not able to gain access to needed resources for persons who were seeking them for well-being, let alone for a MAiD consult.

## Discussion

This is one of the first studies, to our knowledge, to report on physician and nurse practitioner MAiD assessor and provider experiences of Bill C-7 after the legislation was passed. Bill C-7 required participants to navigate regulatory and systems recalibration, including contending with legal uncertainty, inadequate guidance and new regulatory and systems processes. This reflects other studies in Canada after Bill C-14, and internationally, which evidence calibration during MAiD implementation.^18,27–30^ Although our results highlight challenges (such as a desire for more information and guidance), implementing Bill C-7 was smoother than after Bill C-14, as practitioners could leverage their existing knowledge, established care coordination systems (where they existed), and professional supports including CAMAP. This process of calibration is still evolving with the release after these interviews of CAMAP guidance for assessing persons with complex chronic conditions^31^, a Model Practice Standard and Advice to the Profession (published by Health Canada)^26^ and a national Canadian MAiD Curriculum (developed by CAMAP and funded by Health Canada).^32^ This suggests that some gaps identified in this study are starting to be addressed.

Strong participant consensus was that Bill C-7 addressed some key problems under Bill C-14, such as overly stringent witnessing requirements and the 10-day waiting period for Track 1 patients. Participants reported considerably more complexity regarding the waiver, Track 2 and the future permissibility of MAiD MD-SUMC, reflecting previous anticipatory studies.^18,19,33^ However, while some were reluctant or refused to take on Track 2 cases (for a range of contextual and personal reasons which reflect other literature^18,19,34^), the majority (31/32) of participants supported the inclusion of patients with a non-RFND in principle. This disconnect between in-principle support for Track 2 and hesitancy to participate suggests that to foster patient access more work is needed to support practitioners willing to take on Track 2 cases. Similar reservations about participation were also reported for MAiD MD-SUMC and will need to be addressed before the sunset clause takes effect in March 2024.

This research also describes the unexpected impacts of Bill C-7. The waiver was more complex to implement than participants had predicted, with challenges in interpretation and application that echo Variath *et al*.’s^19^ anticipatory study. Despite these challenges, participants emphasized evolving comfort with the waiver over time, with reliance on CAMAP and peers to navigate this. For Track 2, participants indicated significantly less cases came forward than were expected, which was a surprise and may suggest that Track 2 has not disrupted the existing MAiD system to the extent participants in Pesut *et al*.’s^18^ anticipatory study feared. Nevertheless, the variety of Track 2 cases was unexpected with many involving very complex clinical situations such as multiple chemical sensitivities and chronic pain, especially with an overlay of mental illness. Echoing findings by Wiebe *et al*.,^20^ such cases particularly engaged concerns about access to appropriate health care and social services, which participants in this study indicated have no easy answers.

### Implications for regulatory systems

This study has implications for supporting ongoing regulatory changes in Canada, including the implementation of MAiD MD-SUMC. Other jurisdictions considering legalization may also use these findings to help improve implementation. We highlight five key implications.

First, the unanticipated challenges from Bill C-14 provide a lesson to engage practitioners in designing legislative changes, implementing them and evaluating how they are operating in practice. Practitioners apply a clinical lens and can identify unclear aspects of a proposed regulatory framework, which legislative provisions will be difficult to implement (e.g. RFND), and what unintended consequences may and do arise once the legislation is passed. As part of this, a planned implementation period prior to the legislation taking effect is critical; the implementation period for the *Act Respecting End-of-Life Care* in Quebec,^9^ the sunset clause for MAiD MD-SUMC and the 15- to 18-month planned implementation periods in Australian states^35^ are key examples.

Second, Bill C-7 is a good example of legislators addressing what practitioners (and others) identified as unintentional barriers to access. The 10-day waiting period and overly stringent witnessing requirements caused unnecessary access problems for some patients. Likewise, the final consent waiver (known as ‘Audrey’s Amendment’) was introduced after advocacy by Audrey Parker, a woman who chose to die by MAiD sooner than she wished because of concerns about losing capacity.^9^ Similarly, removing RFND eliminated a barrier to access and brought the MAiD legislation in line with the *Carter* decision and the *Charter of Rights and Freedoms*.^10^ These changes were welcomed by participants, and several noted this process of evidence-based regulatory adjustment was appropriate and desirable, and not a ‘slippery slope’.

Third, this study underscores the need to appropriately support MAiD assessors and providers. MAiD practice can be challenging (although rewarding),^33,36^ and this study highlighted how Bill C-7 introduced a greater diversity and complexity of cases. MAiD systems should support practitioners through adequate remuneration, guidance, practice standards, forms and mechanisms for professional and peer support. These supports are particularly important for the logistically and ethically challenging aspects of Bill C-7; the higher the level of anxiety or uncertainty with practice the more important guidance for practice and peer support becomes. This guidance and support enhances consistency in practice and leads to greater clinician confidence and willingness to participate (a key part of system sustainability as demonstrated in this study and others).^29,33^ This support is particularly important to combat what Frolic and Oliphant label the ‘looming access crisis’, with the number of new MAiD providers not increasing proportionally with the number of cases.^37^

Fourth, a broader (but related) point is the importance of integration, coordination and support for the MAiD system as a whole. While assessors and providers are central to MAiD provision, this study also emphasizes the importance of adequately resourced MAiD teams comprised of care coordination, clinical leadership and allied health support. As other research has found, multidisciplinary care pathways are integral to a patient-centred approach.^38,39^ Given the complexity of Track 2 MAiD (including especially Track 2 MAiD MD-SUMC), and the diverse practice settings in which MAiD is delivered (hospital programs through to community delivery), this may require different strategies depending on the geographic and care context.^18^ In parallel, although data suggest it is not socially vulnerable cohorts who are getting MAiD,^15–17,40^ governments should strive to increase support and services responding to forms of structural vulnerability, including disability and mental health care.

Finally, this study raises the question of who should lead the work of engaging with and supporting MAiD assessors and providers, and resourcing and supporting the broader system. An obvious challenge highlighted by this study is the difficulty of implementing MAiD in a system that has responsibilities for both federal and provincial/territorial governments. However, the modern regulatory theory also recognizes regulation is polycentric^41^ and that other non-government organizations will also seek to guide behaviour, and do so. Important examples identified in this study are CAMAP, provincial Colleges and non-governmental organizations such as Dying with Dignity (which in addition to its advocacy, works to support MAiD implementation through initiatives such as education and its volunteer witness program). This raises the question though of how these various bodies operate together to guide MAiD practice. For example, as noted, who should lead MAiD practice and regulation, including coordinating and/or engaging with other bodies in the field. Even if others are involved in regulation, governments must recognize their duty to provide and coordinate regulation to support safe and accessible MAiD.

### Limitations

This study has several limitations. First, Canada is a vast country with ten provinces and three territories, each with responsibility for MAiD service delivery. These findings only reflect the experiences of the 32 MAiD assessors and providers in the three provinces studied. Nevertheless, given the selected provinces provided geographic and regulatory variability, the findings may still be instructive for other areas. Furthermore, the sample had strong internal validity, with coherence in the sample. External validity is also demonstrated by strong coherence with previous anticipatory/early studies on Bill C-7^18–20^ and on the calibration required post-Bill C-14.^27–29^

Second, data were collected from October 2021 to October 2022, 7–19 months after Bill C-7 was enacted and prior to the sunset clause for MAiD MD-SUMC taking effect. Although it is a strength to capture early practice, a limitation is that this research reports on new aspects of MAiD practice in a state of flux. For example, there were only 219 Track 2 cases in 2021 across Canada (2.2% of total cases)^16^ and therefore participants had only limited personal experience of providing Track 2 cases, reflecting evolving MAiD practice. Further research is needed once MAiD MD-SUMC is implemented. Additionally, research with other key stakeholders, including patients, family caregivers, care coordinators and clinicians (including allied health professionals) who are not MAiD assessors and providers, would provide further perspectives on the impact of Bill C-7.

## Conclusion

This study provides insight into the early experiences of MAiD assessors and providers with Bill C-7, which took effect in March 2021. It highlights the complexity of providing MAiD for persons who have a non-RFND. Nearly all participants supported Track 2 MAiD in principle but recognized and experienced challenges with their level of comfort, logistics and burdens of practice in this challenging area. Given the complexities of healthcare delivery across Canada, this study illustrates the importance of adequate support for both practitioners and the system as a whole. While the research showed some areas of inadequate guidance, it also found leadership, dedication and resilience from MAiD assessors/providers, leveraging experience from Bill C-14 and drawing on support from peers, CAMAP and other sources. As a lesson for other jurisdictions, this research illustrates how polycentric regulation functions and shows the need for strong leadership and coordination by government in implementing MAiD into the healthcare system.

## Supplemental Material

sj-pdf-1-pcr-10.1177_26323524231218282 – Supplemental material for Practitioners’ experiences with 2021 amendments to Canada’s medical assistance in dying law: a qualitative analysis Click here for additional data file. Supplemental material, sj-pdf-1-pcr-10.1177_26323524231218282 for Practitioners’ experiences with 2021 amendments to Canada’s medical assistance in dying law: a qualitative analysis by Eliana Close, Jocelyn Downie and Ben P. White in Palliative Care and Social Practice

sj-pdf-2-pcr-10.1177_26323524231218282 – Supplemental material for Practitioners’ experiences with 2021 amendments to Canada’s medical assistance in dying law: a qualitative analysis Click here for additional data file. Supplemental material, sj-pdf-2-pcr-10.1177_26323524231218282 for Practitioners’ experiences with 2021 amendments to Canada’s medical assistance in dying law: a qualitative analysis by Eliana Close, Jocelyn Downie and Ben P. White in Palliative Care and Social Practice

## Declarations

**Ethics approval and consent to participate:** Ethics approval was provided by the Dalhousie University Research Ethics Board (REB #2021-5688 and #2020-5313) and Queensland University of Technology Human Research Ethics Committee (UHREC #2000000270). This research was conducted in accordance with the requirements of this ethics approval. All participants gave free and informed consent to take part in this research.

**Consent for publication:** Not applicable.

**Competing interests:** BPW was contracted by the Victorian, Western Australian and Queensland governments to provide the legislatively mandated training for health practitioners involved in voluntary assisted dying in those states. EC was employed on these projects. BPW is a member of the Queensland Civil and Administrative Tribunal, the quasi-judicial review body that has jurisdiction over some voluntary assisted dying matters. JD was part of several initiatives that contributed to MAiD law reform in Canada: the Royal Society of Canada Expert Panel: End-of-Life Decision-Making; the plaintiff’s pro bono legal team in *Carter v Canada* (*Attorney General*) [2015] 1 SCR 331; the Provincial-Territorial Expert Advisory Group on Physician-Assisted Dying and the Council of Canadian Academies Expert Panel on Medical Assistance in Dying. JD was also a member of a Working Group that developed a module for the federal MAiD National Curriculum (funded by Health Canada and developed by the Canadian Association of MAiD Assessors and Providers) and a member of the MAiD Practice Standard Task Group (mandated by Health Canada to develop a model practice standard for the regulation of MAiD by provincial/territorial health professional regulatory bodies). JD is also on the Advisory Board for the Completed Life Initiative, a national MAiD advocacy and education initiative in the United States. All views expressed in this article are those of the authors and not the organizations they are affiliated with.

**Availability of data and materials:** The interview guide is available in Supplemental Material 2. Due to confidentiality undertakings given to research participants as a requirement of the study’s ethical approval, the data generated and analysed in this study are not publicly available. Requests to discuss this should be directed to the corresponding author.
