# Supplementary material for: Practitioners’ experiences with 2021 amendments to Canada’s medical assistance in dying law: a qualitative analysis
Source: Palliat Care Soc Pract. 2023 Dec 25;17:26323524231218282. doi: 10.1177/26323524231218282 (PMC10750527; doi:10.1177/26323524231218282)
Supplement: sj-pdf-1-pcr-10.1177_26323524231218282 – Supplemental material for Practitioners’ experiences with 2021 amendments to Canada’s medical assistance in dying law: a qualitative analysis [file sj-pdf-1-pcr-10.1177_26323524231218282.pdf]

Supplementary file 1. COREQ Checklist

Canadian practitioners' experiences with 2021 amendments to medical assistance in dying (MAiD) laws: a qualitative analysis

Eliana Close[1], Jocelyn Downie[2], Ben P White[1]

[1] Australian Centre for Health Law Research, Faculty of Business and Law, Queensland University of Technology, Brisbane, Australia

[2] Health Law Institute, Faculties of Law and Medicine, Dalhousie University, Halifax, Canada

Item and guide questions/description	Response	Location in manuscript (section)
**Domain 1: Research team and reflexivity**		
Personal Characteristics		
1. Interviewer/facilitator: Which author/s conducted the interview or focus group?	EC	Methods
2. Credentials: What were the researcher's credentials? E.g., PhD, MD	PhD (Law), MA (Law), BSc (Honours) (Psychology)	-
3. Occupation: What was their occupation at the time of the study?	At the time of the study, EC was a Senior Research Fellow at Queensland University of Technology.	-
4. Gender: Was the researcher male or female?	Female	-
5. Experience and training: What experience or training did the researcher have?	EC has training and experience in law and psychology. She has extensive previous experience in conducting qualitative interviews.	-
Relationship with participants		
6. Relationship established: Was a relationship established prior to study commencement?	Prior to the interview, participants were contacted by EC to provide the participant information and consent form. EC answered participants questions about the study (when they arose) prior to them providing informed consent.	Methods
7. Participant knowledge of the interviewer: What did the participants know about the researcher?	Participants were informed that EC was conducting this research as part of a Canadian case study nested within a broader international comparative study.	Methods
8. Interviewer characteristics: What characteristics were reported about the interviewer/facilitator?	Participants were informed that EC's background was in psychology and in law, and that she was working as postdoctoral research fellow in Australia. They were	-

	also informed that she was a Canadian, originally from the province of Alberta.	
**Domain 2: Study design**		
Theoretical framework		
9. Methodological orientation and theory: What methodological orientation was stated to underpin the study? e.g. grounded theory, discourse analysis, ethnography, phenomenology, content analysis	In-depth semi-structured qualitative interviews were conducting using reflexive thematic analysis.	Methods
Participant selection		
10. Sampling: How were participants selected?	Through convenience sampling (based on calls for recruitment distributed on social media and via professional networks) and subsequently using purposive and snowball sampling.	Methods
11. Method of approach: How were participants approached?	Participants were approached via email and through calls on social media.	Methods
12. Sample size: How many participants were in the study?	32 participants were interviewed.	Results
13. Non-participation: How many people refused to participate or dropped out? Reasons?	None of the participants dropped out of the study.	-
Setting		
14. Setting of data collection: Where was the data collected?	Interviews were conducted using Zoom videoconferencing software.	Methods
15. Presence of non-participants: Was anyone else present besides the participants and researchers?	No	-
16. Description of sample: What are the important characteristics of the sample?	Participants were medical assistance in dying (MAiD) assessors/providers from British Columbia, Ontario, and Nova Scotia. Sample characteristics are reported in the manuscript and in Table 3.	Methods, Table 3
Data collection		
17. Interview guide: Were questions, prompts, guides provided by the authors? Was it pilot tested?	The semi-structured interview guide is available as Supplementary File 2. On request, participants were provided with an overview of prompts prior to the interview. JD was present for three initial interviews and the interview guide was refined with her input.	Methods

18. Repeat interviews: Were repeat interviews carried out? If yes, how many?	No	-
19. Audio/visual recording: Did the research use audio or visual recording to collect the data?	Digital audio- and video-recordings of all interviews were made using Zoom videoconferencing software. The video was immediately deleted to protect participant privacy. Audio-recordings were transcribed verbatim.	Methods
20. Field notes: Were field notes made during and/or after the interview or focus group?	Yes	Methods
21. Duration: What was the duration of the interviews or focus groups?	Interviews ranged from 50 to 203 minutes (median 98.5 minutes).	Results
22. Data saturation: Was data saturation discussed?	Recruitment continued until there was adequate "information power", i.e. sufficient data breadth and depth to meet the study aims.	Methods
23. Transcripts returned: Were transcripts returned to participants for comment and/or correction?	Yes	Methods
**Domain 3: Analysis and findings**		
Data analysis		
24. Number of data coders: How many data coders coded the data?	EC coded the data and refined the coding structure through discussions with JD and BW.	Methods
25. Description of the coding tree: Did authors provide a description of the coding tree?	No	-
26. Derivation of themes: Were themes identified in advance or derived from the data?	As this study is part of a broader investigation, EC identified material relating to Bill C-7 then coded these extracts inductively.	Methods
27. Software: What software, if applicable, was used to manage the data?	NVivo was used for thematic analysis (reported in the manuscript). Microsoft Excel was used for demographic data.	Methods
28. Participant checking: Did participants provide feedback on the findings?	No	-
Reporting		
29. Quotations presented: Where participant quotations presented to	Yes	Results

illustrate the themes/findings? Was each quotation identified?		
30. Data and findings consistent: Was there consistency between the data presented and the findings?	Yes	Results
31. Clarity of major themes: Were major themes clearly presented in the findings?	Five themes are reported in the results and described in Table 4.	Results, Table 4
32. Clarity of minor themes: Is there a description of diverse cases or discussion of minor themes?	Minor themes and description of diverse points of view are discussed in the manuscript.	Results
